# Parkinson’s disease models and death signaling: what do we know until now?

**DOI:** 10.3389/fnana.2024.1419108

**Published:** 2024-10-29

**Authors:** Luiz Fernando A. T. Pedrão, Pamela O. S. Medeiros, Estela C. Leandro, Barbara Falquetto

**Affiliations:** Department of Pharmacology, Instituto de Ciências Biomédica, Universidade de Sao Paulo, Sao Paulo, Brazil

**Keywords:** Parkinson’s disease (PD), cell death mechanisms, apoptosis, neural control of breathing, autophagy, PD animal models

## Abstract

Parkinson’s disease (PD) is the second neurodegenerative disorder most prevalent in the world, characterized by the loss of dopaminergic neurons in the Substantia Nigra (SN). It is well known for its motor and non-motor symptoms including bradykinesia, resting tremor, psychiatric, cardiorespiratory, and other dysfunctions. Pathological apoptosis contributes to a wide variety of diseases including PD. Various insults and/or cellular phenotypes have been shown to trigger distinct signaling events leading to cell death in neurons affected by PD. The intrinsic or mitochondrial pathway, inflammatory or oxidative stress-induced extrinsic pathways are the main events associated with apoptosis in PD-related neuronal loss. Although SN is the main brain area studied so far, other brain nuclei are also affected by the disease leading to non-classical motor symptoms as well as non-motor symptoms. Among these, the respiratory symptoms are often overlooked, yet they can cause discomfort and may contribute to patients shortened lifespan after disease diagnosis. While animal and *in vitro* models are frequently used to investigate the mechanisms involved in the pathogenesis of PD in both the SN and other brain regions, these models provide only a limited understanding of the disease’s actual progression. This review offers a comprehensive overview of some of the most studied forms of cell death, including recent research on potential treatment targets for these pathways. It highlights key findings and milestones in the field, shedding light on the potential role of understanding cell death in the prevention and treatment of the PD. Therefore, unraveling the connection between these pathways and the notable pathological mechanisms observed during PD progression could enhance our comprehension of the disease’s origin and provide valuable insights into potential molecular targets for the developing therapeutic interventions.

## 1 Introduction

Parkinson’s Disease (PD) is a most recognized syndrome, clinically identifiable by a progression of motor and non-motor symptoms, such as bradykinesia, rigidity, akinesia, dystonia, dysphagia, cognitive impairments, and impairments in gut function and olfaction, among others (Poewe et al., 2017; Simon et al., 2020). Symptoms most commonly begin in elderly people (above 60 years), with a prevalence ranging from 1 to 2 per 1,000, rising to more than 4% in those over 85 years of age. This establishes PD as the second most common neurodegenerative disease worldwide (Tysnes and Storstein, 2017; de Rijk et al., 1995; Simon et al., 2020; Aarsland et al., 2021). Although PD is most prevalent in older populations, it is not absent in individuals younger than 61 years, the average onset age of the disease (Pagano et al., 2016). Recent studies indicate that while the incidence of PD is lower in younger individuals, there is a trend towards the development of the disease even in populations traditionally considered to be of lowered risk (Willis et al., 2022). The etiology of PD remains a topic of debate, as it has been shown that both genetic factors, such as mutations in SNCA (Polymeropoulos et al., 1997) and Parkin (Kitada et al., 1998), and environmental exposure, such as the pesticides paraquat and maneb (Cicchetti et al., 2005), may cause the disease (Kouli et al., 2018). The most prominent mutations associated with the increased risk of developing PD are in the GBA and LRRK2 genes. Certain populations, such as the Ashkenazi Jews and North African Imazighen, have shown an increased number of PD cases associated with mutation in these genes (Clark et al., 2007; Hulihan et al., 2008; Healy et al., 2008; Benamer and de Silva, 2010; Ross et al., 2011; Dagan et al., 2015). Other studies have demonstrated significant correlation between these genetic variants and more genetically diverse populations, such as in Brazil (dos Santos et al., 2010; Guimarães Bde et al., 2012) and well as PARK1 mutation in Filipinos (Rogaeva et al., 2004).

Although much remains to be discovered, the primary theoretical pathway through which Parkinson’s disease spreads was hypothesized by Braak and colleagues (Braak et al., 2003). This theory suggests that a pathogen may trigger the progression of the disease by initiating the production of α-synuclein aggregates. This process is thought to occur in two neuron-populated sites: the olfactory bulb and the gut, which may explain the early development of olfactory and gut dysfunctions that precede the motor symptoms defining the diagnosis (Rietdijk et al., 2017; Kim et al., 2019; Konings et al., 2023; Espinosa-Oliva et al., 2024). This hypothesis has led to the establishment of stagings of sporadic Parkinson’s disease based on the presence of α-synuclein aggregates throughout the nervous system (Braak et al., 2003). Stage 1 is characterized by lesions in the dorsal IX/X motor nucleus and/or the intermediate reticular zone; Stage 2 involves additional lesions in caudal raphe nuclei, gigantocellular reticular nucleus, and coeruleus–subcoeruleus complex; Stage 3 includes midbrain lesions, particularly in the pars compacta of the substantia nigra (SN); Stage 4 adds prosencephalic lesions, with cortical involvement limited to the temporal mesocortex (transentorhinal region) and allocortex (CA2-plexus), while the neocortex remains unaffected; Stage 5 sees the involvement of high-order sensory association areas of the neocortex and prefrontal neocortex; and Stage 6 involves lesions in first-order sensory association areas of the neocortex and premotor areas, with occasional mild changes in primary sensory areas and the primary motor field (Braak et al., 2003).

Regardless of the primary cause of the disease, there is broad consensus that the cellular mechanisms involved in cell death, along with inflammation and oxidative stress, play key roles in its development (Tansey et al., 2022; Subramaniam and Chesselet, 2013; Dionísio et al., 2021). In this review, we discuss the role of cell death mechanisms and explore current frontiers in research, both in humans and animal models, including potential treatment opportunities.

## 2 Cell death in neurodegenerative diseases

Although apoptosis is often regarded as a deleterious process, it is essential for developmental tissues to activate apoptosis under certain conditions to remodel tissue or form specific developmental structures (Phelan et al., 1997; Dong et al., 2015; Yamaguchi and Miura, 2015; Voss and Strasser, 2020). When genes related to the initiation of apoptosis are deleted, developing tissue cannot form properly, leading to neurodevelopmental issues such as spina bifida, improper neural tube closure, deficient removal of interdigital webs, and other tissue malformations (Kuida et al., 1996; Cecconi et al., 1998; Yoshida et al., 1998; Ke et al., 2018; Fogarty et al., 2019). In adults, however, apoptosis also contributes to the development of the neurodegenerative diseases through axonal degeneration and neuronal cell death (Pemberton et al., 2021). Numerous studies have shown that the apoptotic pathway is involved in the pathogenesis of these diseases, and it is activated only as a last resort when there is no possibility of neurons recovery (Chi et al., 2018; Dailah, 2022).

Neurodegenerative diseases are characterized by the slow, progressive loss of neuronal cells in the central nervous system (CNS) and the aggregation of misfolded proteins (Cenini et al., 2020). The most common neurodegenerative diseases, such as Alzheimer’s (AD), Huntington’s (HD) and PD, are all marked by the accumulation of misfolded proteins, which play a crucial role in the dysfunction or loss of neurons through their deposition within cells or the extracellular matrix (Singh et al., 2019). While the composition and location of these aggregates can vary between different neurodegenerative diseases, a higher concentration of these proteinaceous materials is generally associated with more severe disease progression (Singh et al., 2019). Although protein aggregation is a common feature, neurodegenerative diseases exhibit different patterns of neurons loss and affect distinct regions of the CNS (Dugger and Dickson, 2017).

Research efforts have led to the creation of animal and cellular models that are useful for unraveling many of the causes of neurodegenerative diseases. However, these models have significant limitations. Some models can display certain molecular or behavioral hallmarks of PD while failing to replicate others. For example, no rodent models that can replicate all the common behavioral symptoms of PD, which limits the choice of animal models depending on the specific behavioral trait being studied (Deumens et al., 2002). Additionally, gene regulation and expression may not bind to the same genes or even chromosomes, leading to different cellular responses to the model’ stimuli (Wilson et al., 2008). The main challenge, however, lies in modelling a disease as heterogeneous as PD, which can present cellular and molecular hallmarks differently between individuals, despite appearing similar among patients (Bloem et al., 2021). The underlying mechanisms of Parkinson’s disease seem to arise from a complex interplay of abnormal α-synuclein aggregation, mitochondrial and lysosomal dysfunction, disruptions in vesicle and synaptic transport, and neuroinflammatory processes (Bloem et al., 2021). To advance our understanding of PD, it is important to clearly define which aspects of the disease we aim to explore and how our research question aligns with the chosen model.

## 3 Mechanisms by which cells can die under various physiological and pathological conditions

### 3.1 Apoptosis

Apoptosis, notoriously known as a type of programmed cell death, is an energy-dependent cellular process that promotes cell death by activating endonucleases and proteases, which ultimately destroy cell molecules. This process leads to biochemical modifications within the cell, rendering it nonfunctional, which can deteriorate tissues and contribute to the development of various diseases (Elmore, 2007). More recent studies describe apoptosis as a regulator of cell fate, determining which cells should be eliminated due to DNA mutations or other proteins malfunctions and which cells should be preserved to maintain homeostasis. This process is largely dependent on the BCL-2 family of proteins (Singh et al., 2019). An imbalance between the pro-death and pro-survival proteins (also known as pro-apoptotic and anti-apoptotic proteins, respectively) can trigger downstream proteins in this pathway, leading to DNA fragmentation and cell death. Additionally, external signals, such as phosphatidylserine, when recognized by nearby phagocytic cells, stimulate the phagocytosis of the apoptotic debris, resulting in a non-inflammatory cell death (Nagata et al., 2016).

Apoptosis operates through two distinct pathways: the intrinsic pathway, which is dependent on internal signaling, primarily regulated by the BCL-2 family of proteins and involves mitochondria interactions, and the extrinsic pathway, which is dependent of external signaling and independent of BCL-2 and mitochondrial involvement (Jan and Chaudhry, 2019). Although these pathways involve different proteins, they converge on the same downstream effectors, known as the execution pathway (Belizário et al., 2015). In Parkinson’s Disease (PD), a sizable portion of neuronal cell death is attributed to apoptosis, as brains from human with PD, as well as those from animal models, exhibit abnormal protein profiles in regions such as the SN, hippocampus, hypothalamus, olfactory bulb and other areas (Erekat, 2018). Indeed, classical literature shows that apoptosis is a common mechanism by which cells respond to well-described apoptotic stimuli, including the drugs traditionally used to induce PD models in both animals (Mendez and Finn, 1975; Heikkila et al., 1984; Perese et al., 1989; Ferrante et al., 1997; Thiffault et al., 2000) and cells (Hartley et al., 1994; Mochizuki et al., 1994; Walkinshaw and Waters, 1994). During cellular respiration and ATP production in mitochondria, reactive oxygen species (ROS) are naturally produced in the electron transport chain (Subramaniam and Chesselet, 2013). However, when ROS are produced uncontrollably, they became toxic to the cell, generating oxidative stress that leads to cell death through apoptosis (Gorman et al., 1996). This type of mitochondrial dysfunction is one of the contributing factors to neurodegeneration in PD (Yan et al., 2013).

Despite evidence showing that apoptosis is a crucial mediator of neuronal death in PD, other cell death mechanisms should not be overlooked. Indeed, animal models exhibiting increased apoptotic signaling may also activate other signaling pathways that contribute to overall degeneration in the brain, such as autophagy (Garcia-Garcia et al., 2013; Zhu H. et al., 2023; Elesawy et al., 2024; Sophoronea et al., 2024), pyroptosis (Zhang M. et al., 2020; Zhu et al., 2022; Huang et al., 2024) and necroptosis (Roy et al., 2023; Kim et al., 2023; Leem et al., 2024). A wealth of research has explored these pathways in the context of Parkinson’s disease, as discussed below.

#### 3.1.1 Intrinsic pathway

**FIGURE 1:**
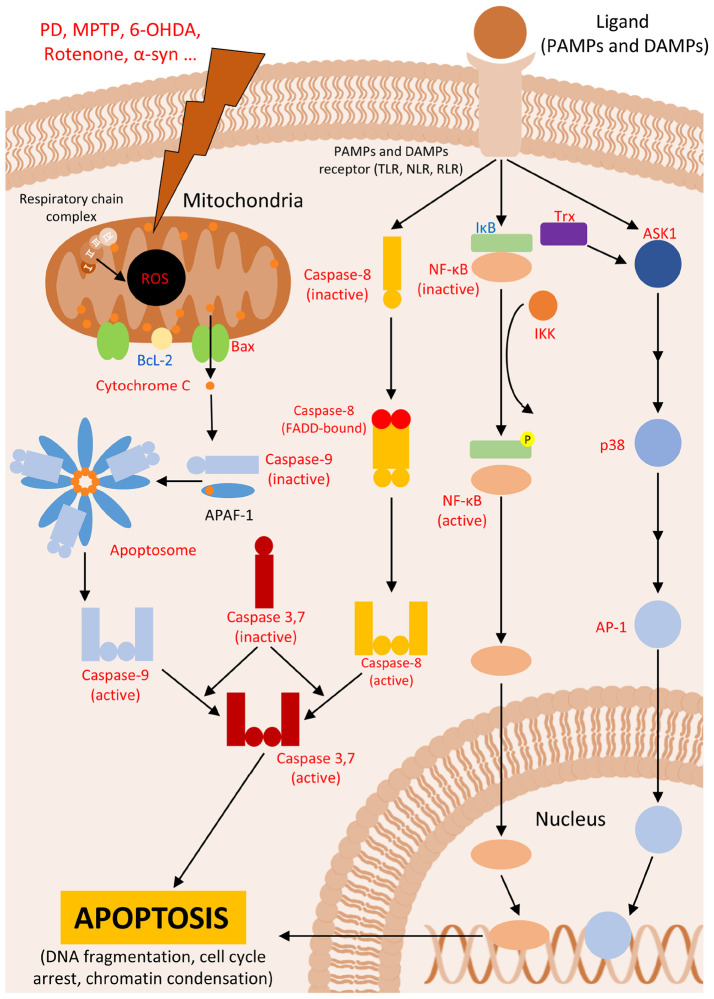
Apoptosis. Figure shows intrinsic and extrinsic pathways leading to apoptosis in Parkinson’s disease (PD) or PD animal models. In red are described upregulated- and in blue downregulated proteins in PD.

The intrinsic pathway is a well-characterized pathway, in which cell fate is regulated through the expression of proteins containing the BH3 domain, such as Bcl2, Bcl-XL, Bax, Bak, Bid, PUMA and NOXA (Alberts, 2022). These proteins are located on the mitochondrial membrane or in the cytosol of the mitochondrial. They play a critical role in maintaining cell survival. Upon activation of the intrinsic pathway, pro-apoptotic proteins are activated, allowing the passage of cytochrome C from the mitochondria, and initiating the apoptotic cascade (Morris et al., 2021; Figure 1). In PD animal models, Bcl-2 family proteins exhibit abnormal expression following neuronal lesions, which triggers apoptotic cascades and contributes to further degeneration of brain regions (Table 1). Reestablishment the balance between these proteins is crucial for returning the tissue to homeostasis (Rekha and Selvakumar, 2014; Liu et al., 2018). Additional studies have highlighted the involvement of both Bax and caspase 3 in PD neurodegeneration across various models, including SH-SY5Y cells (Itano and Nomura, 1995), PC12 cells (Blum et al., 1997), human post-mortem tissue (Tatton, 2000) and mice (Yamada et al., 2010).

**TABLE 1:** Association between apoptotic intrinsic pathway and Parkinson’s disease.

Pathway target	PD model	Action	Timepoint	References
Bcl-2 and Bax	MPTP-induced C57BL/6 mouse model and KO of Bcl-2, TMEM175 and Bax in HEK293T cells	Physical interaction between Bcl-2 and TMEM175 generated ROS and promoted mitochondrial disruption, leading to SN dopaminergic neurons death and motor impairments in mice	7d after daily MPTP injection; Mice of ages ranging from 2w to 18mo to access KO	Qu et al., 2022
Bax, Bcl-2 and cleaved-caspase 3	MPTP-induced C57BL/6 mouse model.	Treatment with isoalantolactone (IAL) can restore basal level of apoptotic proteins disrupted by the MPTP-model induction and ameliorate motor output in animals.	Treatment with IAL started 3d before MPTP injection on mice	He et al., 2022
Cyt-C, cleaved caspase-3, cleaved caspase-9 Bax, Bcl-2	6-OHDA-induced Sprague-Dawley (SD) rat model; 6-OHDA-induced PC12 cell model.	Inhibition of NHE1 improved motor output in SD rats, as well as restored cytochrome C levels in cytoplasm, restored Bax/Bcl-2 balance and reduced ROS production in cells.	After injection of 6-OHDA, rats were treated for 3w with NHE1 inhibitor; cells were treated with NHE1 siRNA 12h prior to 6-OHDA.	Xing et al., 2021
Bax, Bcl-2, caspase 3 and NLRP3	MPTP-induced C57BL/6 mouse	CBD activity was able to restore balance between Bax and Bcl-2 and reduce the activity of both apoptosis and pyroptosis.	MPTP injection for 7d and treated with CBD for 14d	Wang L. et al., 2022
Bax and Bcl-2	Rotenone-induced C57BL/6 mouse, SK-N-SH and primary cells	PLG prevents the imbalance in apoptosis caused by rotenone disruption of mitochondrial permeability.	After 6w of administration with rotenone, PLG or L-dopa was administered for 4w	Liu et al., 2018
Bax	MPP+ model of SH-SY5Y cells	miR216a interacts directly with Bax and ameliorates the apoptotic signaling in cells, including reduction in caspase activity.	Treatment with miR-216a 4h-48h prior to MPP+	Yang x. et al., 2020
Bax and BcL-2	6-OHDA-induced Wistar male rats	Decrease of Bcl-2 and increase of Bax in respiratory neurons leading to its degeneration after PD model induction	30d after PD induction with 6-OHDA	Falquetto et al., 2020
Caspase-3	6-OHDA-induced Wistar male rats	Stress worsens the levels of caspase-3 in comparison to the 6-OHDA model	Restraint stress started 7d post-injection of 6-OHDA	Idrissi et al., 2023

Following the release of cytochrome C, it interacts with Apaf1, exposing its CARD domain, which allows this protein to bind to other Apaf1 proteins, forming an oligomer known as the apoptosome (Dorstyn et al., 2018). The apoptosome the assembles initiator caspases, such as caspase 8 and 9 (Bao and Shi, 2007; McIlwain et al., 2013; Anson et al., 2021). These initiator caspases, once activated, lead to the activation of effector caspases (typically, caspases 3, 6, and 7) through the cleavage of the latter. These effector caspases are responsible for cleaving the cell’s DNA, thereby completing the apoptosis signaling cascade (Brentnall et al., 2013; Parrish et al., 2013; Figure 1). Research by Fall and Bennett indicates that apoptosis in SH-SY5Y cells induced with MPTP begins 9 to 12 hours after induction, during which ROS production continues and mitochondria membrane potential is lost, leading to apoptosis (Fall and Bennett, 1999).

Several factors can lead to the uncontrolled production of ROS, including damage to the mitochondrial complex I and III, which are the major sources of ROS, reduced ATP production, or malfunctioning of enzymes such as superoxide dismutase (SOD) that convert ROS into non-toxic molecules (Elfawy and Das, 2019). Postmortem analyses of brains from PD patients reveal colocalization between cytochrome c and other apoptosome-related proteins with Lewy bodies, highlighting the role of these proteins in the formation of such structures (Kawamoto et al., 2014). Dopaminergic neurons in the SN are particularly sensitive to oxidative stress (Sziráki et al., 1998) due to their elevated levels of pro-oxidant iron, which facilitates ROS production by reducing oxygen, and their low levels of glutathione, a crucial antioxidant in cellular metabolism (Sian-Hülsmann et al., 2011). Previous study has demonstrated a correlation between ROS production, as indicated by SOD activity, and PD models (Choi et al., 1999).

#### 3.1.2 Extrinsic pathway

As its name suggests, the extrinsic pathway is triggered primarily by extracellular signals mediated by immune system cells, such as lymphocytes or macrophages. These cells produce soluble molecules, including members of the tumor necrosis factor superfamily (TNFSF), which diffuse through the tissue and bind to their receptors on target cells, leading to the progression of apoptosis (Yanumula and Cusick, 2023). Another extracellular mechanism for triggering cell death involves the interaction of receptors and ligands, such as the Fas/FasL system, which can also initiate apoptotic events under certain conditions (Yamada et al., 2017). Receptors involved in the extrinsic pathway form complexes with caspase 8, triggering apoptosis though the terminal pathway, which is also associated with the intrinsic apoptotic pathway (Medema et al., 1997; Bodmer et al., 2000; Figure 1). The activation of caspase 8 as an apoptotic inducer, has been well-described in PD *in vitro* models (Viswanath et al., 2001; Choi et al., 2004), along with the involvement of proteins from other proteins from the extrinsic pathway (Table 2).

**TABLE 2:** Association between apoptotic extrinsic pathway and Parkinson’s disease.

Pathway target	PD model	Action	Timepoint	References
TNFα; α-synuclein	SH-SY5Y cells; TNFα homozygous-KO mice injected with α-synuclein	TNFα was able to induce cell senescence and cell-to-cell α-synuclein propagation via secretion of the protein	Evaluated after 12w of α-synuclein injection	Bae et al., 2022
TNFα; IL-1β	6-OHDA-induced rat model	Presence of IL-1β in rats’ serum was correlated with moderate lesion of SN DAergic neurons, whilst presence of TNFα in rats’ serum was correlated with advanced lesion of SN DAergic neurons.	Analysis was performed before, after 2 and 8w of 6-OHDA injection	Piri et al., 2022
TNFα; IL-1β	6-OHDA-induced C57BL/6 mouse model	L-DOPA is responsible of inducing dyskinesia (LID) in animals with partial lesion of SN, increasing plasmatic levels of TNFα and IL-1β, and treatment with CBD + CPZ lowered TNFα levels and ameliorated the LID and inflammatory prognostic of the model.	Treatment with L-DOPA started 3w after 6-OHDA injection and lasted until the 45^th^ day; CBD+CPZ was used from the 42^nd^ to the 45^th^ day.	dos Santos Pereira et al., 2021
Fas-FasL; FOXO3, PUMA	PD patients’ serum; 6-OHDA SH-SY5Y cell model	miR128 is downregulated in PD patients’ sera, and its supplementation on 6-OHDA-treated cells can reestablish balance in apoptotic pathways, reducing FasL and PUMA expression, increasing FOXO3a expression and reducing caspases 3, 8, and 9 activities.	Transfection started 2d after plating of SH-SY5Y cells.	Bhattacharyya et al., 2022
Trx1, ASK1, cyt-c, p38, caspase 3, NF-κB	MPP+-induced SH-SY5Y cells	Through the ASK1/Trx1/p38 pathway, SAL was able to inhibit cell death induced by the MPP+ model, reducing caspase 3 activity, NF-κB expression, and reducing DNA fragmentation.	Cells were treated previously with SAL for 24h and exposed to MPP+ for 48h.	Yang et al., 2021
p-p38	6-OHDA induced Wistar male rats	Decrease in p-p38 and Bax levels in the respiratory retrotrapezoid nucleus after neurodegeneration	30d after PD induction with 6-OHDA	Falquetto et al., 2020
JNK, p38, caspase 3	6-OHDA induced SH-SY5Y cells and C. elegans	NCS was able to diminish 6-OHDA lesion in SN neurons of the nematodes, and it regulated JNK-p38 pathway, inhibiting apoptosis	Cells were treated with NCS for 24h prior to 6-OHDA	Fu et al., 2022
TLR2, NF-κB and IL-1β	C57BL/6 mice seeded with preformed α-synuclein fibrils (PFF)	Blockade of TLR2 receptor using TIDM/NBD is essential to prevent cell death and increase in NF-κB and IL-1β.	Treatment started 2mo after PFF injection	Dutta et al., 2021

The interaction between ligand and receptor proteins triggers intracellular responses involving proteins such as NF-κβ (Rickert et al., 2011), and JNK (Chang et al., 2006), or in some cases, no protein activation due to decoy receptors (MacFarlane et al., 1997). The role of NF-κβ in PD cellular models is well-documented, particularly concerning the neuroprotective effects of blocking this pathway (Cassarino et al., 2000; Huang et al., 2018; Meng et al., 2023; Figure 1).

To better understand the role of these proteins in apoptosis, it is crucial to comprehend how these pathways induce cell death. NF-κβ is a dimer, composed of proteins from the NF-κβ family, which is normally bound to IκBα, which sequesters the dimer in the cytoplasm (Jacobs and Harrison, 1998). NF-κβ proteins are produced through the proteolytic processing of two other precursor proteins, p100 and p105. This processing, which involves the cleavage of the C-terminal half of the protein, results in the formation of either NF-κβ2 or NF-κβ1, respectively (Lin and Ghosh, 1996; Yamada et al., 2000). Upon activation of TNFRSF and other inflammatory receptors, a protein complex known as IKK phosphorylates IκBα, targeting it for degradation by the proteasome. This action releases NF-κB, allowing it to translocate to the nucleus and function as a transcription factor, in what is known as the canonical pathway (Liu et al., 2017; Figure 1).

Additionally, these receptors can activate mitogen-activated protein kinases (MAPK), a superfamily of proteins known for their role in phosphorylating other proteins on serine and threonine residues. This phosphorylation leads to signaling cascades that can activate gene transcription through the complex formation between receptor and the mitogen (Gómez and Cohen, 1991). Some members of the MAPK family, such as JNK and p38, despite their key role in cell survival via the activation of growth factors, are also involved in apoptosis. These proteins can also be activated by receptors responsible to stress stimuli and inflammatory cytokines (Cano et al., 1994; Cano and Mahadevan, 1995), and their activation is sufficient to trigger apoptosis in PD models (Onyango et al., 2005; Ouyang and Shen, 2006). It has been demonstrated that deprivation of certain nutrients to cells *in vitro* (e.g., tropic stimuli, glucose, ions) can activate alternative pathways that lead to a detrimental activation of JNKs, promoting apoptosis (Xu et al., 2001; Wilms et al., 2003; Song and Lee, 2007; Ramiro-Cortés and Morán, 2009). Moreover, there are evidence that p38 pathways can trigger NF-κβ translocation, further exacerbating degeneration in dopaminergic neurons of the SN in PD animal models (Karunakaran and Ravindranath, 2009; Yan et al., 2017). Furthermore, ROS contribute to the apoptotic pathway mediated by Trx-ASK1 and p38 in microglia, as Trx is an oxidative stress-sensitive marker that can trigger this pathway to regulate cell death (Noguchi et al., 2008; Hirata et al., 2020).

The cascade leading to apoptosis involves the activation of the transcription factor AP-1. Depending on the combination of proteins such as c-jun, c-fos, and others, AP-1 can regulate target genes that determine cell fate through mechanisms such as cell cycle progression, arrest, or apoptosis (Lee et al., 1987; Ameyar et al., 2003). The apoptotic activation of AP-1, leading to cell death, can be delayed by the expression of Bcl-2, which underscores the interaction between JNK/p38 pathways and intrinsic apoptosis (Bossy-Wetzel et al., 1997). Research has shown that p38 interacts with components of the intrinsic pathway and p53 (Perfettini et al., 2005; Farley et al., 2006), and this interaction is also observed in PD models (Karunakaran et al., 2008; Chen et al., 2018; Chen et al., 2020; Figure 1).

Finally, an important apoptosis activation pathway involves various stress signals that triggers the JNK/p38 pathway and leads to apoptosis. One of the most common stress signals in PD animal models and other oxidative-dependent diseases is the oxidative stress. In this context, sensor proteins such as Ask1/Trx are activated, which in turn activate JNK/p38, leading to apoptosis (Hsieh and Papaconstantinou, 2006; Pan et al., 2010; Hu et al., 2011; Yamada et al., 2012).

### 3.2 Necroptosis

Previously thought of as an unregulated and uncoordinated form of cell death, necrosis (or necroptosis) has been identified as an alternative, regulated pathway of cell death, primarily dependent on the tumor necrosis factor receptor 1 (TNFR1) and its ligand, TNF? Research has also shown that this pathway can induce apoptosis (Laster et al., 1988; Gao et al., 2024; Kazmi et al., 2024). This association has been noted since the 1990’s, with studies showing increased levels of TNFα and its type 1 receptor in the SNC of PD patients (Boka et al., 1994; Mogi et al., 1994; Sriram et al., 2002). Other extrinsic apoptotic mechanisms, such as Toll-like receptors (TLR) and Fas/FasL, can also trigger necrosis. Despite the different pathways leading to cell death, there is a significant crosstalk between them, which depends on the cellular and tissue context, such as after PD lesion stimuli in animal models (Hartmann et al., 2001; Oberst et al., 2011; Kaiser et al., 2011; Zhang et al., 2011; Table 3).

**TABLE 3:** Association between necrosis pathway and Parkinson’s disease.

Pathway target	PD model	Action	Timepoint	References
RIPK1; TNFα; IL-1β;	MPTP-induced C57BL/6 mice; MPP^+^-induced SH-SY5Y cells	Inhibiting RIPK1 enabled animals to have a better motor output and demonstrate lower levels of TNFα and IL-1β, which was also improved by inhibiting the ASK1-p38-JNK pathway.	Nec-1s treatment for 1mo, 5d after MPTP; 12h of Nec-1s incubation after MPP+	Liu et al., 2021
TNF receptor 1	6-OHDA induced male mice	Degeneration of brainstem respiratory areas were prevented by TNFR1 knockout in male mice model of PD	10d after PD induction with 6-OHDA	Cabral et al., 2024
RIPK1; FADD; caspase 8 and caspase 9	HEK293T cells induced with A53T- α-synuclein	A53T- α-synuclein was able to increase protein levels of RIPK1, FADD, altogether with caspase 8/caspase 9 activity, in contrast with WT- α-synuclein, increasing apoptosis	30 min of exposure to A53T- α-synuclein	Meshkini et al., 2023
RIPK1; MLKL; TNFα; IL-1β; α-synuclein	MPTP-induced C57BL/6 mouse model	Treatment with NSA ameliorated motor output of animals, and reduced phosphorylation of MLKL, preventing aggregation of MLKL with α-synuclein, and reduced expression of IL-1β, TNFα, and iNOS	NSA treatment for 20d, starts the day after the 5^th^ daily injection of MPTP	Leem et al., 2023
RIPK1; MLKL.	6-OHDA-induced C57BL/6 mouse model; C57BL/6 primary mesencephalic neurons induced with 6-OHDA	Axon degeneration in cell culture was abrogated by inhibiting necrosis using nec-1s, also ameliorating motor output in mice.	Concomitant treatment of Nec-1s with exposure to 6-OHDA	Oñate et al., 2020
RIPK1; RIPK3; MLKL; NF-κB	Primary midbrain human astrocytes; SH-SY5Y neurons induced with fibrillar α-syn	PFF-treated cells presented higher expression of necroptotic proteins that were required to demonstrate higher levels of NF-κB.	24h exposure of cells to PFF, and treatment for 30 min with necrosis inhibitors	Chou et al., 2021
Zn^2+^	Wistar male rats injected with NMDA and AMPA	Animals injected with AMPA, simulating excitotoxicity in DA neurons, were treated with a fluorescent zinc probe, showing that zinc influx was a reflex of cell stimulation by AMPA, enhancing ROS signaling in cells, which was reverted by ROS-capturing agents	After 30 min of AMPA injection, treatment with the Zn^2+^ probe was performed for 20 min.	Tamura et al., 2023

**FIGURE 2:**
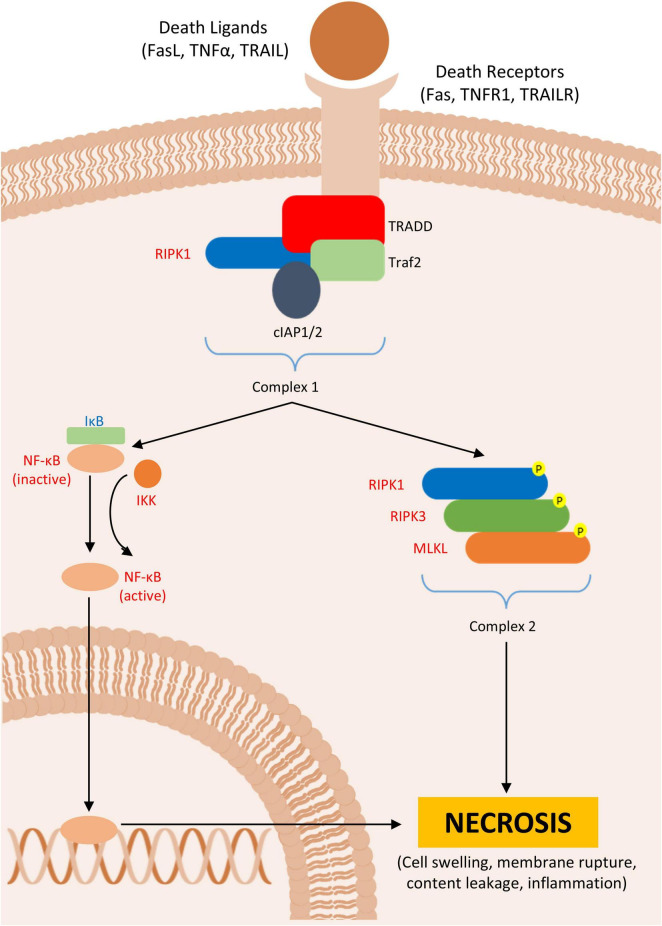
Necroptosis. Figure shows the formation of ripoptosome, which leads to necroptosis in Parkinson’s disease (PD) or PD animal models. In red are described upregulated- and in blue downregulated proteins in PD.

In general, these receptors interact with their ligands, and most of these pathways respond to their stimuli inducing NF-κβ gene transcription. However, some stimuli (e.g., pharmacological agents) can inhibit either RIPK1 or caspase 8 activity, thereby favoring the activation of necrosis pathways *in vivo* (Lin et al., 1999; Thapa et al., 2013). There are three key phenomena essential for the mechanism of necrosis: (1) the recruitment of RIPK1 to the TNFR1 by the adaptor protein TRADD, followed by the recruitment of TRAF2, another adaptor protein, which then associates with cIAP1/2, leading to a reduced caspase activation; (2) the recruitment and phosphorylation of RIPK3 by this membrane complex; and (3) the recruitment of mixed-lineage kinase-like protein (MLKL), which, upon phosphorylation by the RIPK1-RIPK3 complex, assembles into a new complex, called the necrosome (Sun et al., 2012; Chen et al., 2013; Weber et al., 2018; Faergeman et al., 2020; Figure 2).

Necrosis is also observed in PD models. Inhibition of necrosis using necrostatin-1, a potent inhibitor of RIPK1, is associated with reduced dopaminergic cell death in the SN in both *in vivo* and *in vitro* models (Wu et al., 2015; Iannielli et al., 2018). Moreover, the formation of protein complexes associated with TNFR1 through RIPK1 has been observed in screenings of necrotic and apoptotic regulators. Genes associated with this protein aggregation have been correlated with the development of PD and other neurodegenerative diseases (Amin et al., 2018). It has been noted that the loss of cell integrity, associated with necrosis is an important hallmark of MPTP and rotenone PD cell models, with elevated expression of RIPK3 (Callizot et al., 2019).

Although studies have shown that inhibition of MLKL can reduce microglia activation and, consequently, inflammation (Lund et al., 2005; Geng et al., 2023), the presence of the necrosome cluster alone is not sufficient to initiate necrosis. MLKL is a self-inhibited protein; it requires binding of other proteins to expose its active domain and promote its migration to the plasma membrane, thereby completing the necroptotic pathway. This underscores the highly regulated nature of necrotic cell death (Dovey et al., 2018; McNamara et al., 2019). Upon activation, the MLKL complex migrates to the plasma membrane, where its accumulation forms hotspots that open ion channels, causing cell swelling, membrane rupture, and the formation of pores. This results in the extrusion of intracellular contents and subsequent necrosis (Yoon et al., 2014; Samson et al., 2020; Liu et al., 2024; Table 3).

Bioinformatic studies have shown that genes associated with necroptosis are altered in the brains of PD patients compared to control subjects, suggesting an increased susceptibility to necrotic cell death in these patients (Lei et al., 2023). Interestingly, investigations into the genetic variance risk related to intrinsic inhibition of TNFα or TNFR1-TNFα have found no correlation between the age of onset of PD and inhibition of this pathway, indicating that further research is needed to understand the role of necrosis in disease development (Kang et al., 2021). In PD models, evidence suggest that ablation of necroptosis effectors can attenuate inflammation and necrosis caused by neuroinflammation driven by agents like LPS or MPTP (Geng X. et al., 2023; Kim et al., 2023).

A particular form of necrosis, known as excitotoxicity, is caused by excessive stimulation of neurons by neurotransmitters (Choi, 1992). Increased activation of receptors, such as NMDAR and AMPAR, leads to an influx of ions like Ca^2+^, through the membrane. This increase ion concentration interacts with the endoplasmic reticulum, which in turn activates calpain, enhances ROS production and ultimately disrupts cell function. The disruptions can result in cell lysis, mitochondrial dysfunction, and organelle destruction (Brorson et al., 1995; D’Orsi et al., 2012; Gupta et al., 2013; Zhou et al., 2013; Polster et al., 2022). The relationship between excitotoxicity and development of PD has been extensively discussed (Ilijic et al., 2011; Pan et al., 2017; Soman et al., 2019)

### 3.3 Pyroptosis

**FIGURE 3:**
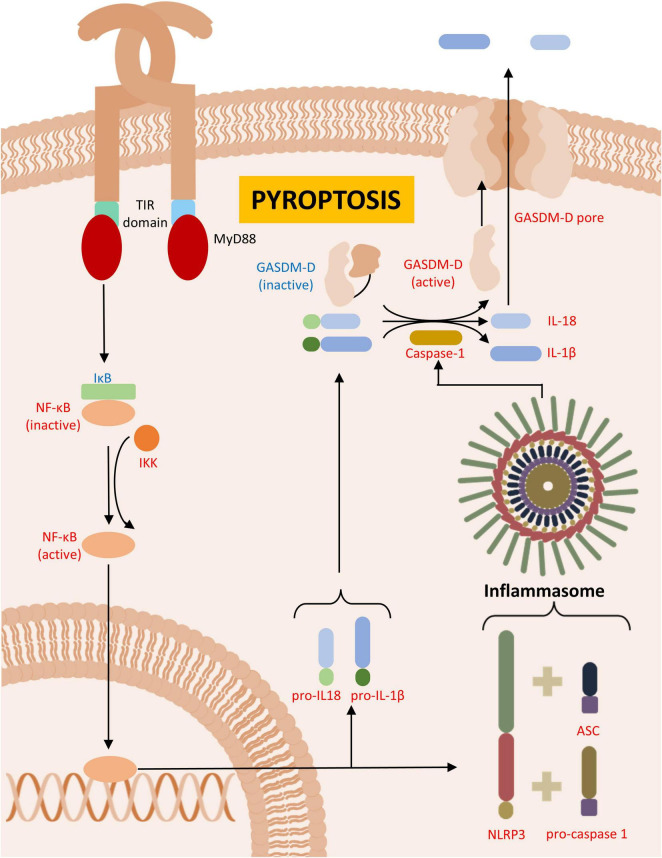
Pyroptosis. Figure illustrates the formation of inflammasome, leading to pyroptosis in Parkinson’s disease (PD) or PD animal models. In red are described upregulated- and in blue downregulated proteins in PD.

Similar to necrosis, pyroptosis is a form of inflammatory, coordinated cell death. However, unlike necrosis, pyroptosis was initially described as a type of programmed cell death that, rather than being quiet, like apoptosis, triggers a robust inflammatory response, including the recruitment of immune cells to the site of death (Boise and Collins, 2001; Cookson and Brennan, 2001). A key characteristic of pyroptosis is the conversion of interleukin-1β (IL-1β) and interleukin-18 (IL-18) via caspase 1 (Thornberry et al., 1992). IL-1β is implicated in the neurodegeneration observed in PD as it can exacerbate neuroinflammation and promote the death of dopamine neurons, underscoring the significance of pyroptosis in PD (Koprich et al., 2008; Codolo et al., 2013; He et al., 2015; Table 4). Throughout the process of pyroptosis, caspase 1 is responsible for cell swelling (Fink and Cookson, 2006), DNA fragmentation (Bergsbaken and Cookson, 2007), the arrest of cell metabolism (Shao et al., 2007), and other related functions (Figure 3).

**TABLE 4:** Association between pyroptosis and Parkinson’s Disease.

Pathway target	PD model	Action	Timepoint	References
Caspase 1, ASC, NLRP3, IL-1β	MPTP-induced C57BL/6 mice; 6-OHDA-induced SH-SY5Y cells	Dl-3-n-butylphthalide (NBP) can prevent neurodegeneration and motor deficits in animals through reducing NLRP3-associated pyroptosis and mitochondria impairments, with reduction in caspase 1, ASC, and IL-1β, also seen in cells	NPB treatment started at the 5^th^ day of MPTP injection, and lasted 14d	Que et al., 2021
Caspase 1, ASC, NLRP3, IL-1β	MPTP-induced C57BL/6 mouse; MPP+-induced N9 mice microglia	MPP+ and LPS were sufficient to trigger inflammasome activation both in culture and in animals, and treatment with Andro was able to restore motor function and inflammasome-related protein levels to basal	14d of Andro after MPTP injection; 1h after MPP+	Ahmed et al., 2021
TNFα; IL-1β; NF-κB; Bcl-2; Bcl-xL; Bax; PUMA; caspase 3; caspase 9	MPTP-induced C57BL/6 mouse	Besides the improvement of motor output, celastrol was also able to prevent alterations on the transcription of mRNA related to cell death signaling, and its mechanisms is related to the activation of the NLRP3-caspase1 axis through Nrf2.	4d of MPTP injection, and in the 5^th^ day, treatment with celastrol went on for 5d.	Zhang et al., 2021
Parkin; NLRP3; ASC; GSDM-D caspase 1	Parkin^flx/flx^ mice; Casp1^–/–^ / parkin^flx/flx^ mice	Ablation of parkin was sufficient to cause degeneration of SN neurons in mice, with increase in NLRP3, ASC, GSDM-D expression, and caspase 1 activity, showing increased inflammasome activity.	Cre-recombinase injection in 6-8w old mice, and test were performed 3mo after that.	Panicker et al., 2022
NLRP3; ASC; IL-1β; caspase 1; α-synuclein	C57BL/6 mice seeded with preformed α-synuclein fibrils (PFF); MitoPark mice.	MCC950 leads to a decreased formation of α-synuclein-activated inflammasomes and using this molecule in animal models helped inhibiting α-syn aggregation, ameliorating the motor deficits, and improving cell survival through reduction in the expression of inflammasome-related proteins.	Treatment with MCC950 started before injection of 6-OHDA	Gordon et al., 2018
NLRP3; ASC; caspase-1; GSDM-D; IL-1β; IL-18; TLR4; NF-κB	MPTP-induced C57BL/6 mouse; MPTP-induced C57BL/6 TLR4-deficient mouse; PC12 and BV2 MPP^+^-induced cells	SAL ameliorates mice motor output and reduced inflammasome- and inflammatory-related proteins, through direct interaction with MyD88 and NLRP3	SAL treatment started after 5d of MPTP injection, and lasted 5d.	Zhang X. et al., 2020
IL-1β; IL-18; NLRP3; GSDM-D; caspase 1; ASC	MPTP-induced C57BL/6 mouse; BV2 MPP+-induced cells.	BHB arrests pyroptosis inhibiting STAT3/NLRP3/GASDM pathway and ameliorates motor deficits caused by the MPTP model	BHB treatment for 56d; MPTP injection was done from the 42^nd^ to the 49^th^ day of BHB treatment.	Jiang et al., 2022
p38; NF-κB; NLRP3; caspase 1; ASC; IL-1β	6-OHDA-induced Sprague Dawley rats; BV2 LPS -induced cells	Both KAE and SB203580 were able to ameliorate pyroptosis by suppressing the p38 pathway both in animals and in BV2 microglia.	Starting from the 2^nd^ w after 6-OHDA injection, KAE was injected for 30d.	Cai et al., 2022
NLRP3; NLRP1; caspase 1; Nrf2	6-OHDA-induced Wistar rats with Nrf2 overexpression; 6-OHDA induced PC12 cells transfected with Nrf2	Nrf2 overexpression was able to increase AABR07032261.5 expression both in animals and in cells, which in turn reduced expression of inflammasome-related proteins and decreased cell death, improving motor behavior in animals.	Nrf2 plasmid injection 3 w prior to 6-OHDA induction; 48h treatment with Nrf2 plasmid after 24h induction with 6-OHDA	Zhong et al., 2022

The initiation of pyroptosis depends on the sensing of the extracellular microenvironment, primarily through TLRs (Nyström et al., 2013) or the cytosolic space via NOD-like receptors (NLRs) (Qiu et al., 2017). TLRs are responsible for detecting pathogen-associated molecular patterns (PAMPs) and damage-associated molecular patterns (DAMPs) produced by cell death and pathogen elimination (Srikrishna and Freeze, 2009; Naqvi et al., 2022). Upon activation, the main pathway involved in the inflammatory responses is mediated by the production of cytokines such as TNFα, IL-6, IL-1β and IL-12 (Ozato et al., 2002; Cantó et al., 2006; Covacu et al., 2009; Rodriguez et al., 2019). This pathway relies on an intracellular domain, TIRAF, which recruits adapter proteins such as MyD88, triggering NF-κβ-regulated genes and modulating inflammatory responses (Bonnert et al., 1997; Zheng et al., 2019; Figure 3). Additional to this pathway, there are other pathways that involve different adaptor proteins, but ultimately lead to the activation of NF-κβ and other inflammatory signaling molecules, such as AP-1 (Yamamoto et al., 2002; Honda and Taniguchi, 2006).

NLRs can detect PAMPs and DAMPs and subsequently produce inflammatory cytokines regulated by NF-κβ (Abbott et al., 2004). However, certain NLRs belong to a specialized group that can form intracellular protein complexes known as inflammasomes, which contribute to ongoing pyroptosis and cytokines production – these are the NLRP receptors (Pétrilli et al., 2007; Hayrabedyan et al., 2016). For the inflammasome to assemble, the NLRP must interact with caspase 1-derived components, especially IL-1β (Martinon et al., 2002). It is known that the interaction of dopamine with NLRP3 can inhibit pyroptosis, further highlighting the relationship between this cell death mechanism and PD (Yan et al., 2015).

These receptors possess intracellular domains responsible for interacting with other proteins and protein complexes, such as CARDs bound to caspase 1, or using their pyrin domain to recruit CARDs – an essential stage for cytokines production and the binding of the adapter protein ASC (Boucher et al., 2018; Yang et al., 2019). In PD pathology, ASC specks are considered hallmarks of pyroptosis, as their expression has been observed in peripheral blood mononuclear cells, and this exacerbation is sufficient to enhance NLRP3 inflammasome formation (Fan et al., 2020; Zheng et al., 2023). The aggregation of multiple ASC specks forms the inflammasome, which role, through the action of bound caspase 1, is to promote cytokine processing. Moreover, by processing of gasdermin D (GDSM) into its active form, the inflammasome facilitates the inclusion of pores in the cell membrane, leading to cell lysis (Ding et al., 2016; Liu et al., 2016; Faria et al., 2021; Figure 3).

Conversely, drugs capable of inducing NLRP3 can establish PD-like models in animals (Wang Y. et al., 2022). Finally, other activation mechanisms contribute to the activation of the NLRP3 inflammasome, further aiding in the development of PD models in animals (Huang et al., 2024; Quan et al., 2024).

### 3.4 Autophagy

Autophagy, unlike the other pathways discussed here, is typically associated with cellular processes that promote cell survival. It plays a crucial role in energy conservation by recycling proteins and other cellular components to meet the energetic demand of cells (Yamamoto et al., 2023). Originally described by Christian de Duve in 1963 as a process involving lysosomes and their enzymes to degrade cellular components, autophagy is now understood to be as far more complex event that is intricately regulated by genetics, pathology, and other factors (Levine and Kroemer, 2008; Klionsky, 2008). There are three types of autophagy, each differing in morphology and mechanism, but leading to the degradation of cellular components within lysosome (Parzych and Klionsky, 2014).

**FIGURE 4:**
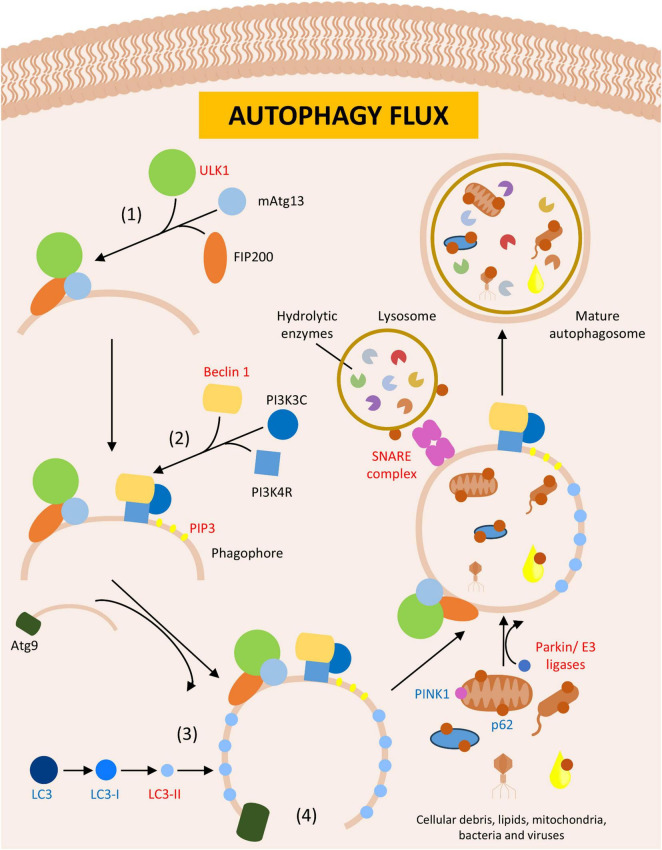
Autophagy. Figure shows the formation of autophagosome leading to dysregulation in autophagy flux in Parkinson’s disease (PD) or PD animal models. In red are described upregulated- and in blue downregulated proteins in PD.

The most studied type of autophagy is the macroautophagy, which involves the formation of bilipid membrane layer called an autophagosome. This structure engulfs organelles and proteins to be degraded, encloses the debris within the vesicular space, and degrades the proteins by fusing the autophagosome with a lysosome (Yi and Tang, 1999; Anding and Baehrecke, 2017; Figure 4). The formation of the autophagosome requires the expansion of its membrane, a process driven by autophagy-related genes known as Atgs (Takeshige et al., 1992). In both lower and higher eukaryotes, the initiation of autophagy requires the assembly of specific protein complexes: (1) the Atg1-Atg13-Atg17 (ULK1-Atg13-FIP200 in mammals) kinase complex, which initiates phagosome formation at the phagophore assembly site (PAS, an Atg8-rich cytoplasmatic site) (Kabeya et al., 2005; Chang and Neufeld, 2009); (2) the phosphatidylinositol 3-kinase complex 1, composed of vacuolar protein sorting proteins such as Beclin 1, PI3KR4, and PI3KC3 in mammals, responsible for the nucleation phase by directing proteins to the PAS through the generation of phosphatidylinositol 3-phosphate (PIP3) (Kihara et al., 2001; Reidick et al., 2017; Iershov et al., 2019); (3) ubiquitin-like assembly complexes, such as Atg12 and Atg8 (LC3 in mammals), which facilitated membrane elongation (Williams et al., 2009; Zhang et al., 2022); and (4) Atg9, a protein involved in the cycling of lipids between the autophagosome and other membranous structures like vesicles, aiding in protein enclose within the autophagosome and assisting in the elongation phase (Reggiori et al., 2005; Yen et al., 2007; Figure 4). After protein enclosure and autophagosome completion, lysosomes interact with this structure to degrade the autophagosome contents, a process mediated by SNARE complexes, which facilitate membrane fusion (Söllner et al., 1993; Hu et al., 2003; Hong, 2005; Jahn and Scheller, 2006).

Research on PD patients has shown abnormal expression of proteins involved in autophagy (Table 5) and Atg and ULK genes, which increased upstream regulators of autophagy and decreased downstream regulators, strengthening the link between the disease and this cellular mechanism (Miki et al., 2018). In mammals, LC3 is a widely observed gene related autophagy dysfunctions and serves as a marker of autophagy activity in cells (Kabeya et al., 2000). Additionally, Beclin1 is associated with autophagy and other vesicle transport pathways and plays an important role in the interplay between autophagy and other cell death mechanisms, as discussed further in this article (Liang et al., 1998; Kang et al., 2011; Lõrincz et al., 2014; Tran et al., 2021). Lastly, p62 is a protein that binds to ubiquitinated protein aggregates, cellular debris, bacteria, and viruses, targeting them for the autophagosome. It also interacts with proteins that lead to the autophagy of entire organelles, including the PINK1 protein, a genetic factor in PD, which is involved in mitochondria autophagy (Pankiv et al., 2007; Dagda et al., 2009; Lamark et al., 2009; Clausen et al., 2010; Geisler et al., 2010; Wurzer et al., 2015). Another important protein in PD development is PARK7/DJ1, which is involved in genetic variants of the disease. Its loss increases protein aggregation, overburdens autophagy and mitophagy, alters cellular machinery, and promotes oxidative stress (Krebiehl et al., 2010; Bai et al., 2020; Imberechts et al., 2022).

**TABLE 5:** Association between autophagy pathway and Parkinson’s disease.

Pathway target	PD model	Action	Timepoint	References
Beclin 1; LC3; ULK1	MPTP-induced C57BL/6 mouse; SH-SY5Y cells induced with MPP^+^, transfected with antisense miR-132-5p	Blockade of miR-132-5p influence both on cells and animals can reduce MPTP-related increase in apoptosis and autophagy, and this effect is repeated after overexpression of ULK1, highlighting the interaction between ULK1 and miR-132-5p.	2d of treatment with antisense miR-135-5p or control prior to MPTP model	Zhao et al., 2020
Caspase-1; NLRP3; IL-1β	Mice with Atg5-KO in CX3CR1^+^ (microglia) cells; BV2 cells induced with LPS.	The interaction between Atg5 KO in microglia and MPTP potentializes motor dysfunction and SN neuron death in mice, increasing pyroptosis-related gene expression	MPTP was injected in 10w mice; silencing of Atg5 was done 48h prior to 24h-LPS injection	Qin et al., 2021
Bcl-2; Bax; p62; LC3; Beclin 1	MPTP-induced C57BL/6 mouse, transfected with miR-497-5p antagonist or miR-497-5p antagonist + shFGF2; SH-SY5Y cells induced with MPP^+^, transfected with miR-497-5p mimics or inhibitors	MPP^+^ upregulated miR-497-5p transcription, which in turn upregulated pro-apoptotic related genes and reduced autophagic flux, and inhibition of such micro-RNA restored basal levels of proteins from both signaling cascades.	2d before MPTP injection; transfection of cells was done 1d before MPP+ injection in culture.	Zhu et al., 2021
Bcl-2; Bax; caspase 3; LC3; mTOR; Beclin 1; Atg5	C57BL6 mouse and PC12 cells induced with rotenone (Rot) or PM2.5	Both PM2.5 and Rot were able to disrupt autophagic flux and induce apoptosis, and co-treatment further increased the deleterious effects of treatments.	Cells were treated with rapamycin for 1h before receiving rotenone or PM2.5; animals were treated concomitantly with Rot, PM2.5 and rapamycin for 28d.	Wang et al., 2021
LC3; p62	Hs-SNCA-expressing C57BL/6 mice	Abnormal expression of α-syn in mice results in diminished autophagic flux, which was reestablished by treatment with piperine (PIP).	PIP treatment was administered for 6mo in mice	Li et al., 2022
NLRP3; caspase 1; IL-1β; LC3	MPTP-induced C57BL/6 mice, treated with a CB2R inhibitor; Primary astrocyte culture.	Depletion of CB2R aggravated MPTP model, as activation of CB2R degrades NLRP3 through autophagy and thus shuts down the pyroptosis pathway.	siRNA was injected 4w prior to MPTP injections.	Zhu H. et al., 2023
LC3; Beclin1; p62; AKT	MPTP-induced C57BL/6 mice transfected with BDNF; SH-SY5Y cells induced with MPP^+^, transfected with BDNF	BDNF was able to prevent cell death and enhance autophagy in culture after MPP^+^ lesion, and mice had a better motor output and autophagy-related protein expression increase after treatment with BDNF	BDNF was injected 2d prior to MPTP model establishment.	Geng X. et al., 2023

Another type of autophagy is the chaperone-mediated autophagy (CMA), a highly selective pathway that involves the transportation of specific proteins across the lysosome membrane via a receptor (Dice, 1990; Hubert et al., 2022; Fregno et al., 2018; Loi et al., 2019). In PD models, the role of this gene, along with LRRK2 - another key gene associated with autophagy in PD−has been shown to be crucial in stimulating autophagy and preventing neuronal degeneration (Issa et al., 2018; Ho et al., 2020).

## 4 Autophagy x apoptosis

The literature extensively debates the protective mechanisms involved in autophagy and whether, in neurodegenerative diseases, this mechanism acts as an inhibitor of cell death or, conversely, enhances death signaling and thus promotes cell death. Research indicates that, over the loss of critical transducer molecules within cell, such as MAPKs, allows adaptor proteins of receptors to interact with other proteins, which mobilize certain cell death mechanisms depending on the presence or absence of p62 (Goodall et al., 2016). Furthermore, there is an important connection between the permeabilization of mitochondria outer membrane and autophagy, as p62 serves as a tag regulating PUMA degradation, leading to a reduced apoptosis (Thorburn et al., 2014). More recent studies show that proteins such as mitochondria translation elongation factors are responsible for efficient mitochondrial autophagy (Zhu J. et al., 2023). Blocking these proteins results in caspase-8 activation and increased TNFα sensitivity, thereby enhancing and accelerating apoptosis (Choi et al., 2022). Conversely, autophagy appears to protect against apoptotic cell death by regulating proteins involved in both pathways, including Bcl-2 and Bcl-xL, and Beclin1 (Pattingre et al., 2005; Maiuri et al., 2007). According to Maycotte and colleagues, autophagy may precede apoptosis and is necessary for caspase activation, as seen in increased autophagosome formation and LC3 processing when the cells (rat cerebellar granule neurons) were treated with substances that increase ROS, thereby decreasing the cell viability (Maycotte et al., 2010).

On the other hand, in neurodegenerative diseases, the impaired function of the autophagy system itself appears to be direct consequence of blockage caused by the accumulation of misfolded proteins. This impairment is exarcebated by an increase in ROS, despite autophagy’s critical role in removing these proteins aggregates (Bandyopadhyay and Cuervo, 2007; Janda et al., 2012). Furthermore, the amount of Ca^2+^ released from the endoplasmic reticulum plays an important role in regulating apoptosis, as it signals the mitochondria, modulating autophagy suggesting a complex interplay between these pathways and necrosis, especially excitotoxicity-induced necrosis (Høyer-Hansen et al., 2007; Cárdenas et al., 2010).

However, studies also show that while autophagy may contribute to death signaling in neurons, it simultaneously acts as a mechanism to continuously monitoring the cell’s survival state. Research has described a reciprocal regulation between Atg7 and caspase 9, resulting in an autophagy-dependent apoptosis flux (Han et al., 2014; Ojha et al., 2016). Moreover, the preservation of cell integrity via autophagy is essential for preventing neurodegeneration in dopaminergic neurons through apoptosis, involving a regulatory mechanism linked to the AKT/mTOR pathway (Zhu et al., 2024).

It is known that autophagy is defective in dopaminergic neurons in the SN, as observed in *post-mortem* brains of patients and certain models of PD like 6-OHDA and rotenone-induced models, which show suppression of mTOR, a key enhancer of autophagy (Grassi et al., 2018). In α-synuclein-induced PD mouse model, Zhang and collaborators showed that the administration of caffeic acid prevents the neurodegeneration of dopaminergic neurons in SN and improves behavioral abnormalities by stimulating autophagy through the JNK/BcL2 pathway (Zhang et al., 2019). Moreover, miRNAs have an important role in regulating autophagy-related genes and signaling pathways. When downregulated, these miRNAs are responsible for neuroprotection by either activating protective autophagy or reducing autophagic neuronal cell death (Choi et al., 2016; Sarkar et al., 2022).

## 5 Novel revelations regarding cell death mechanisms in discrete nuclei affected in PD, notably within critical centers like the respiratory system

**FIGURE 5:**
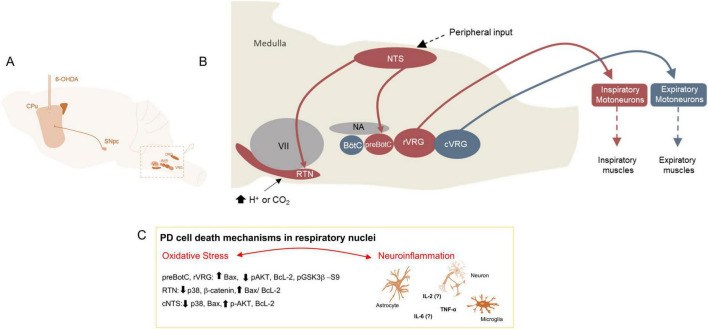
Changes in neural control of breathing in the PD model. **(A)** Figure shows the PD model induction by the injections of 6-OHDA in the CPu leading to degeneration of SN. **(B)** Figure shows the representation of medullary nuclei that are responsible for neural control of breathing. In red are represented nuclei that are degenerated in the PD model. **(C)** Figure shows the mechanisms involved in cell death in the respiratory nuclei. CPu, Caudate-Putamen; SN, Substantia Nigra; VRG, Ventral Respiratory Group; RTN, Retrotrapezoid Nucleus; BötC, Botzinger Complex; preBötC, PreBotzinger Complex; rVRG, Rostral portion of the Ventral Respiratory Group; cVRG, Caudal portion of the Ventral Respiratory Group; DRG, Dorsal Respiratory Group; NTS, Nucleus of the Solitary Tract; NA or Amb, Ambiguus Nucleus; VII: Facial Nucleus.

Current understanding about the role of cell death in Parkinson’s disease is due to neurodegeneration in midbrain, notably within the SN. Although other brain regions have been studied to explore the disease’s impact on the degeneration of unconventional areas, limited insight exists regarding the connection between cell death mechanisms, such as apoptosis, and the neurodegeneration observed in nuclei that govern neural control of breathing. This connection has been previously described alongside functional deficits in the 6-OHDA model of PD (Tuppy et al., 2015; Fernandes-Junior et al., 2018; Oliveira et al., 2019; Figure 5). It is important to note that, since death signaling is not confined to the SN and its related areas, similar signaling may also occur throughout the brain in PD patients and models. Through research is needed to understand the extent of such signaling in these regions. In post-mortem studies of the human brains affected by PD, Benarroch demonstrated that the ventrolateral medulla, which houses the nucleus responsible for respiratory rhythmicity, showed degeneration evidenced by a reduction in NK1 receptors (Benarroch et al., 2003). However, the mechanisms underlying the neurodegeneration of respiratory nuclei in PD remain poorly understood.

Briefly, the neuronal circuitry that controls ventilatory function is located within the medulla oblongata and pons. The pre-Bötzinger Complex (preBötC) is responsible for generating the inspiratory rhythm through pacemaker glutamatergic neurons, which project to the rostral ventral respiratory group (rVRG), a group of pre-motor neurons situated in the ventral region of the medulla (Yang C. F. et al., 2020). The rVRG innervates the diaphragm, initiating its contraction, which expands the thoracic cavity and allows air to enter the lungs (Smith et al., 1991; Vann et al., 2018; Dhingra et al., 2024). Additionally, other nuclei in the pons modulate upper airway muscle activity, creating optimal conditions for air to maximize contact with alveoli, thereby promoting gas exchange. This modulation is also essential for maintaining eupnea and generate various respiratory behaviors (Song et al., 2012; Levitt et al., 2015; Abdala et al., 2016; Farmer et al., 2014; Dutschmann et al., 2021).

The Bötzinger Complex (BötC) and lateral parafacial (pF_*L*_) control the expiration, the final phase of respiration, during which air is expelled from the lungs. This process occurs either through passive relaxation of the diaphragm or active contraction of muscles, enabling CO_2_-rich air to leave the lungs (Huckstepp et al., 2015; de Britto and Moraes, 2017; Zoccal et al., 2018; Silva et al., 2019). Finally, nuclei such as the retrotrapezoid nucleus (RTN) and the nucleus of solitary tract (NTS) play crucial role in sensing or receiving information related to the partial pressure of CO_2_ and O_2_, as well as variations in blood pH, thereby fine-tuning the neural control of breathing (Takakura et al., 2006; de Paula et al., 2007; Del Rio et al., 2012; Ott et al., 2012; Díaz et al., 2020).

Regarding the degeneration of respiratory nuclei, it is known that 30 days after the injection of 6-OHDA in the rat’s CPu, pro-apoptotic signaling occurs in the preBötC and the rVRG. This signaling is characterized by increased intrinsic and extrinsic signaling involving Bax/BcL-2 proteins, leading to the loss of NK1 receptors after 40 days of PD induction, which results in breathing dysfunction (Falquetto et al., 2020). Similarly, in the RTN and NTS, these nuclei experience loss of phox2b^+^ neurons 30 days post-6-OHDA injection. At the same time, they exhibit an anti-apoptotic signaling, as an attempt by the system to recover from the injury. This is demonstrated by a reduction in p38 and Bax and increase of pAKT levels (Falquetto et al., 2020; Aquino et al., 2022; Figure 5). Moreover, oxidative stress is the main candidate responsible for impaired breathing in the PD model, as observed in SN; treatment with apocynin, an antioxidant drug, prevented the neurodegeneration in respiratory nuclei and mitigated respiratory dysfunction in the PD rat model (Nascimento et al., 2022). Lastly, a study has shown the involvement of glial cells and TNF-α in the degeneration of respiratory nuclei and breathing dysfunction in mouse model of PD, underscoring the importance of neuroinflammation (Cabral et al., 2024; Figure 5).

Mechanism under which these neurons may die might connect with neuron’s death Braak’s hypothesis. According to this hypothesis, α-synuclein fibrils spread through the axons in a gut-brain orientation, with the dorsal motor nucleus of the vagus nerve (DMV) (Braak et al., 2003). This dorsal nucleus is known to be connected to other breathing control centers, such as the NTS (Rogers et al., 1980; Kalia and Sullivan, 1982; Davis et al., 2003; Davis et al., 2004), which in turn projects to and receives projections from other respiratory nuclei (Alheid et al., 2011; Yang x. et al., 2020; Biancardi et al., 2021). Conversely, neurodegeneration in brainstem breathing control nuclei might also be explained by the projection of olfactory bulb (OB) neurons to various brain areas. Notably, the OB connects to the NTS via the paraventricular nucleus (Guevara-Guzman et al., 1991), to the locus coeruleus (Shipley et al., 1985; McLean and Shipley, 1991), and to the SN (Höglinger et al., 2015). Moreover, evidence suggests connection between the SN, periaqueductal gray, and the RTN (Lima et al., 2018; Aquino et al., 2022). One might hypothesize the relative contribution of these pathways to the development of degeneration in the respiratory circuitry. However, there is limited understanding of neuronal death in these nuclei in both PD models and human patients, representing a potential area for further research. Overall, these studies underscore the importance of investigating the signaling pathways that lead to cell death in PD models, as these pathways can impair ventilation, potentially affecting the lifespan of animals and, consequently, human health.

## 6 Conclusion

Despite our current understanding of cell death in PD being insufficient to cure the disease, science has advanced considerably since James Parkinson first described it. As the global population ages and the incidence of neurodegenerative diseases increases, it is imperative for biomedical research to better understand the causes and progression of these diseases to improve health outcomes to the elderly. Basic research aimed at elucidating additional factors involved in these pathways is crucial for identifying more target molecules and developing novel therapies that may slow or halt the progression of neurodegenerative diseases. Our comprehension is that as these diseases progress, multiple cellular and tissue mechanisms are recruited to reestablish homeostasis. However, cellular damage often advances faster than the recovery mechanisms can address it, leading to multiple signaling pathways that promote different forms of cell death. This results in tissue damage, and especially on nervous tissue, creates a “dead space” where cells cannot recover due to the lack of neurogenesis. It is important to recognize that while the disease affects the SN neurons and cause the classical symptoms, other brain regions, such as respiratory nuclei, also undergo degeneration. This contributes to symptoms and suffering in patients, highlighting the need for further investigation into the relationship between SN neurons death and the degenerations of other brain areas.

The characteristics of cell death presented here suggest that the molecular aspects most prevalent in PD models can be leveraged to prevent the degeneration of other regions affected during disease progression, potentially extending patient’s lifespan and improving their quality of life. Although a cure for PD has not yet been found, a better understanding of the molecular pathways and genetic variants involved could lead to improve early diagnosis protocols. The protocols could include assessments of patient’s genetic susceptibility and the identifications of better biomarkers to enhance diagnosis accuracy.

Given current hypothesis about the disease’s origins, a promising starting point is to investigate the role of α-synuclein more deeply. It is already established that Lewy’s bodies, which are composed of misfold α-synuclein, contribute to cellular stress that can trigger various forms of cell death (Jiang et al., 2017; Gordon et al., 2018; Ardah et al., 2021; Gao et al., 2022; Bae et al., 2022; Lin et al., 2023; Yildirim-Balatan et al., 2024; Jia et al., 2024). The critical question that remains is whether halting of α-synuclein misfolding and aggregation is enough to stop disease progression or, ideally, to prevent the disease from developing altogether. To address it, it is essential for scientific research to focus on the understanding the underlying causes of PD, the mechanisms of neuronal death, and the identification of new therapeutic targets. By improving early diagnosis and employing available tools to treat patients as soon as possible, we can work towards preventing the establishment of the disease and ensuring a healthier ageing process for the.
